# Case of gastric anisakiasis with no symptoms

**DOI:** 10.1002/ccr3.2948

**Published:** 2020-05-20

**Authors:** Takayuki Yamada, Susumu Ohwada

**Affiliations:** ^1^ Asunaro Clinic Takasaki city Japan; ^2^ ASKOHWADA Consultation Clinic of Gastroenterology and Oncology Maebashi city Japan

**Keywords:** anisakiasis, no abdominal pain, surveillance esophagogastroduodenoscopy, the popularity of sushi and sashimi

## Abstract

We present a unique image from a case of anisakiasis that was detected incidentally in an elderly man during surveillance esophagogastroduodenoscopy. Given the popularity of sushi and sashimi throughout the world, we believe that our observations will raise awareness about the risk of Anisakis contamination in seafood.

## Graphical abstract

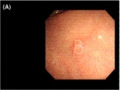

Anisakiasis, a gastrointestinal tract infection associated with acute severe abdominal pain, is caused by consuming raw or undercooked seafood containing Anisakis simplex larvae.^1^ Symptoms generally appear within a few hours. We present an incidentally detected case of anisakiasis in a healthy 82‐year‐old man with no characteristic symptoms during surveillance esophagogastroduodenoscopy (EGD) for chronic atrophic gastritis. EGD revealed a mobile nematode (Figure 1A) burrowing through the mucosal layer of the gastric antrum. No surrounding edema or erythema was observed. The nematode was removed using biopsy forceps (Figure 1B) and histologically confirmed as Anisakis simplex. The patient revealed that he had consumed sashimi (raw young yellowtail) purchased at a supermarket 2 days earlier. Anisakis larvae typically die within 7 days. This report aims to raise awareness about the existence of Anisakis in fish beyond Japan.

**Figure 1:**
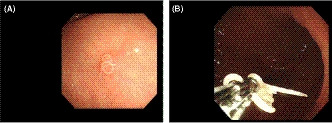
A, Esophagogastroduodenoscopy revealing a mobile nematode. B, Esophagogastroduodenoscopy showing Anisakis larvae removed by biopsy forceps

## CONFLICT OF INTEREST

None.

## AUTHOR CONTRIBUTIONS

TY: served as a diagnostician and first author. SO: served as a supervisory doctor.
